# IL-22 inhibits bleomycin-induced pulmonary fibrosis in association with inhibition of IL-17A in mice

**DOI:** 10.1186/s13075-022-02977-6

**Published:** 2022-12-24

**Authors:** Ziye Qu, Wencan Dou, Kexin Zhang, Lili Duan, Dongmei Zhou, Songlou Yin

**Affiliations:** 1 Department of Rheumatology, The Affiliated Hospital of Xuzhou Medical University, Xuzhou, 221002 China; 2 The First Clinical Medicine School, Xuzhou Medical University, Xuzhou, 221002 China; 3 Blood Diseases Institute, Xuzhou Medical University, Xuzhou, 221002 China; 4 Department of Rheumatology, The People’s Hospital of Jiawang District of Xuzhou, Xuzhou, 221011 China

**Keywords:** IL-22, Bleomycin, Pulmonary fibrosis, IL-17A

## Abstract

**Background:**

Interstitial lung disease, a common extra-articular complication of connective tissue disease, is characterized by progressive and irreversible pulmonary inflammation and fibrosis, which causes significant mortality. IL-22 shows a potential in regulating chronic inflammation and possibly plays an anti-fibrotic role by protecting epithelial cells. However, the detailed effects and underlying mechanisms are still unclear. In this study, we explored the impact of IL-22 on pulmonary fibrosis both *in vivo* and *in vitro*.

**Methods:**

To induce pulmonary fibrosis, wild-type mice and IL-22 knockout mice were intratracheally injected with bleomycin followed by treatments with recombinant IL-22 or IL-17A neutralizing antibody. We investigated the role of IL-22 on bleomycin-induced pulmonary fibrosis and the mechanism in the possible interaction between IL-22 and IL-17A. Fibrosis-related genes were detected using RT-qPCR, western blot, and immunofluorescence. Inflammatory and fibrotic changes were assessed based on histological features. We also used A549 human alveolar epithelial cells, NIH/3T3 mouse fibroblast cells, and primary mouse lung fibroblasts to study the impact of IL-22 on fibrosis *in vitro*.

**Results:**

IL-22 knockout mice showed aggravated pulmonary fibrosis compared with wild-type mice, and injection of recombinant IL-22 decreased the severe fibrotic manifestations in IL-22 knockout mice. In cell culture assays, IL-22 decreased protein levels of Collagen I in A549 cells, NIH/3T3 cells, and primary mouse lung fibroblasts. IL-22 also reduced the protein level of Collagen I in NIH/3T3 cells which were co-cultured with T cells. Mechanistically, IL-22 reduced the Th17 cell proportion and IL-17A mRNA level in lung tissues, and treatment with an IL-17A neutralizing antibody alleviated the severe pulmonary fibrosis in IL-22 knockout mice. The IL-17A neutralizing antibody also reduced Collagen I expression in NIH/3T3 cells *in vitro*. Knockdown of IL-17A with siRNAs or administration of IL-22 in NIH/3T3 cells and MLFs decreased expression of Collagen I, an effect blocked by concurrent use of recombinant IL-17A.

**Conclusions:**

IL-22 mediated an anti-fibrogenesis effect in the bleomycin-induced pulmonary fibrosis model and this effect was associated with inhibition of IL-17A.

**Supplementary Information:**

The online version contains supplementary material available at 10.1186/s13075-022-02977-6.

## Background

Connective tissue disease-related interstitial lung disease (CTD-ILD) is a category of chronic pulmonary disease caused by CTD with progressive and irreversible inflammation and fibrosis which is accompanied by progressive loss of pulmonary function. Occurrence of CTD-ILD indicates poor prognosis in CTD patients [1]. The 5-year survival rate of CTD patients was 43.4% after ILD diagnosis [2], so early diagnosis and timely treatment of ILD can improve the prognosis of CTD patients. Glucocorticoids and immunosuppressants are the primary choices for CTD-ILD patients [3], but the efficacy is limited. Thus, a new therapy is still needed.

ILD is characterized by pathological changes such as diffused pulmonary parenchyma, alveolitis, and interstitial fibrosis. The initial inflammatory response plays a key role in the induction of pulmonary fibrosis through recruiting immune cells and releasing cytokines. Continuous inflammatory response increases fibroblast proliferation and excessive deposition of extracellular matrix, resulting in pulmonary fibrosis [4]. Cytokines and chemokines accelerate the development of fibrosis. For example, TGF-β induces the proliferation and differentiation of fibroblasts and increases the deposition of extracellular matrix [5]; some pro-inflammatory cytokines regulate the development of fibrosis through different molecular mechanisms. IL-8 attracts the migration of macrophages [6]. IL-1β recruits lymphocytes and neutrophils [7]. IL-6 inhibits apoptosis of alveolar type II cells and promotes the proliferation of fibroblasts [8].

IL-22, a member of the IL-10 cytokine family [9], can be secreted by several types of immune cells, in which T cell is a dominant producer. Intriguingly, due to the expression of IL-22 receptor, immune cell-derived IL-22 targets epithelial cells and fibroblasts in tissues such as the respiratory system, digestive system, and skin [10]. IL-22 is a potential target for simultaneously regulating inflammatory and fibrotic responses. IL-22 exerts both anti-inflammatory and pro-inflammatory properties depending on the local microenvironment [11]. Studies have shown that the role of IL-22 in fibrotic diseases is controversial, including a pro-inflammatory function and a tissue-protective effect [12, 13]. IL-22 inhibits hepatic stellate cell apoptosis to alleviate liver fibrosis [14]; IL-22 reduces Collagen I, Collagen III, matrix metalloproteinase-9 (MMP-9), and increases tissue inhibitor of metalloproteinase (TIMP) to improve myocardial fibrosis [15]; IL-22 significantly increased the expression of Collagen I protein without changing its mRNA level in normal human dermal fibroblasts *in vitro* [16]; the mTOR/autophagy pathway in CX3CR1^+^ monocyte phagocytes upregulates the IL-23/IL-22 axis to promote intestinal fibrosis [17]; IL-22 was also reported to reduce pulmonary inflammation and to inhibit bleomycin-induced epithelial-mesenchymal transition, but the underlying mechanism remains unclear [18, 19]. IL-22 is mainly produced by CD4^+^T cells. Th17 cells secrete both IL-17A and IL-22. IL-17A induces the aggregation of inflammatory cells and the release of inflammatory factors, which promotes the development of pulmonary fibrosis, while IL-22 protects epithelial cells and plays an anti-fibrotic role [20, 21]. In this study, we investigated the role of IL-22 on bleomycin-induced pulmonary fibrosis and the possible interaction between IL-22 and IL-17A.

## Methods

### Reagents

Bleomycin was obtained from Solarbio Science & Technology (Beijing, China). Mouse recombinant IL-22 was purchased from STEMCELL Technologies (Shanghai, China). *In Vivo*MAb anti-mouse IL-17A was purchased from Bio X Cell (West Lebanon, NH). Recombinant human TGF-β1 was purchased from PeproTech (100–21, Rocky Hill, NJ, USA). IL-17A(HY-P7372), Mouse (CHO) was from MCE (Monmouth Junction, NJ, USA).

### Mice

Wild-type (WT) C57BL/6 mice (male, 6–8 weeks, weighing 18–22 g each) were purchased from Charles River (Vital River, Beijing, China). IL-22 knockout (KO) mice with C57BL/6 background were purchased from Cyagen Biosciences (Suzhou, China). IL-22KO mice were generated by using Transcription Activator-Like Effector Nuclease (TALEN, Cyagen Biosciences, Suzhou, China). Exon 1 and exon 2 were selected as TALEN target sites. The mRNA transcribed from the targeted allele with frameshift underwent nonsense-mediated decay (NMD). All mice were bred in special pathogen-free rooms. Procedures regarding animal care and experiments were approved by the Experimental Animal Care and Use Committee of Xuzhou Medical University.

### Pulmonary fibrosis model

WT mice and IL-22KO mice were anesthetized with 5% chloral hydrate and injected intratracheally with 100 μl bleomycin at a dose of 5 mg/kg body weight. Untreated normal mice were intraperitoneally injected with PBS, and mice treated with bleomycin were intraperitoneally injected with vehicle, or recombinant IL-22 (200 ng per mouse, 3 times a week for 3 weeks), or IL-17A neutralizing antibody (100 μg per mouse, twice a week for 3 weeks) [22–24].

### Cell culture

The human alveolar epithelial cell line A549 kindly provided by Dr. Dongsheng Pei of Xuzhou Medical University was cultured in DMEM medium (Thermo Fisher Scientific, Waltham, MA, USA) supplied with 10% fetal bovine serum (FBS, Thermo Fisher Scientific). The mouse fibroblast cell line NIH/3T3 cells were kindly presented by the Department of Thoracic Surgery of Xuzhou Medical University cultured in DMEM medium supplied with 10% FBS. For isolating primary mouse lung fibroblasts (MLFs), lung tissues were cut into small pieces followed by a first digestion with 5 mM EDTA, a second digestion with 1 mg/ml collagenase type I plus 0.5 mg/ml collagenase type IV plus 1 mg/ml DNase I, and a third digestion with 0.5 mg/ml dispase plus 1 mg/ml collagenase type I. Isolated primary MLFs were cultured in DMEM medium supplied with 10% FBS. T cells were isolated from spleen cells using a Mouse CD90.2 Positive Selection Kit II from STEMCELL Technologies (Shanghai, China). Isolated mouse T cells were cultured in RPMI-1640 medium (Sigma-Aldrich, Shanghai, China) containing 10% FBS, 3 μg/mL anti-mouse CD3 (145-2C11), and 3 μg/mL anti-mouse CD28 (37.51) (BioLegend, San Diego, CA).

### Isolation of mononuclear cells from lung tissue

For isolating mononuclear cells from lung tissue, samples were cut into small pieces, followed by a first digestion with 5 mM EDTA, a second digestion with 1 mg/ml collagenase type I plus 0.5 mg/ml collagenase type IV plus 1 mg/ml DNase I, and a third digestion with 0.5 mg/ml dispase plus 1 mg/ml collagenase type I. Mononuclear cells were obtained by centrifugation in Lymphocyte Separation Medium (DAKEWE, Shanghai, China).

### Flow cytometry

To analyze helper T cell type-17(Th17) cells, mononuclear cells of lung tissue were incubated with PMA, ionomycin, and Brefeldin A (Sigma-Aldrich) in RPMI-1640 medium for 4 h followed by a process with a Fixation/ Permeabilization Solution Kit (BD Biosciences, San Jose, CA). The processed cells were stained with anti-CD3e (145-2C11), anti-CD4 (RM4-5), anti-IL-17A (TC11-18H10), and anti-IFN-γ (XMG1.2). CD3^+^CD4^+^IL-17A^+^ cells are defined as Th17 subset. The antibodies were purchased from BD Biosciences or BioLegend (San Diego, CA, USA). Cells were acquired and analyzed using an LSRFortessa flow cytometer (BD Biosciences).

### H&E staining and Masson staining

Paraffin sections of lung tissues were used for H&E and Masson staining, and Masson staining was performed with a Trichrome Stain Kit (G1340, Solarbio Science & Technology). The degree of alveolar inflammation and lung injury was assessed by Szapiel’s score[25], Ashcroft score was used to assess the severity of pulmonary fibrosis[26]. The collagen deposition areas were assessed with Image J software.

### Western blotting

Proteins of lung tissues and cells were extracted using RIPA lysis buffer (Beyotime Biotechnology, Shanghai, China). The following antibodies were used: Collagen I (1:1000, 67288–1-Ig), vimentin (1:1000, 10366–1-AP), α-SMA (1:1000, 55135–1-AP), GAPDH (1:5000, 60004–1-Ig) and SMAD3(1:1000, 66516–1-Ig) were purchased from Proteintech. Phospho-SMAD3 (1:1000, AF3362) was purchased from Affinity Biosciences (Cincinnati, OH, USA). Phospho-STAT3 (1:1000, Tyr705) and STAT3 (1:1000, 79D7) were purchased from Cell Signaling Technology (Danvers, MA, USA).

### Quantitative polymerase chain reaction

To detect the expression of mRNA levels, qPCR was performed as described [27]. The primer sequences (Thermo Fisher Scientific Shanghai, China) were provided in Table 1.

**Table 1:** Sequence of primers used in the study

Gene	Forward (5′-3′)	Reverse (5′-3′)
Collagen I	TGACTGGAAGAGCGGAGAGTA	CATTAGGCGCAGGAAGGTCA
Vimentin	TTTGCTGACCTCTCTGAGGC	CTCCAGGGACTCGTTAGTGC
α-SMA	AGCCATCTTTCATTGGGATGG	CCCCTGACAGGACGTTGTTA
IL-17A	CCTGGACTCTCCACCGCAA	TTCCCTCCGCATTGACACAG
IL-22	TCGCCTTGATCTCTCCACTC	GCTCAGCTCCTGTCACATCA
IFN-γ	AGACAATCAGGCCATCAGCAA	GTGGGTTGTTGACCTCAAACT
GAPDH	TTGATGGCAACAATCTCCAC	CGTCCCGTAGACAAAATGGT

### Immunofluorescence

Cryosections of lung tissues were stained with anti-Collagen I (1:100), anti-vimentin (1:100), and anti-α-SMA (1:100). Alexa Fluor 488 goat anti-rabbit IgG (1:300, Invitrogen, Carlsbad, CA, USA) was used as secondary antibodies. Nuclei were counterstained with DAPI (KeyGEN Biotech, Nanjing, China). Images were acquired and analyzed on a Zeiss 880 confocal microscope.

### Cell viability

Cells were incubated with a Cell Counting Kit-8 reagent (CK04, DOJINDO, Tokyo, Japan). Cell viability was determined by measuring optical density at 450 nm.

### Indirect co‐culture

NIH/3T3 cells were indirectly co-cultured with T cells using a Transwell culture system (Corning Incorporated, Corning, NY, USA), which were maintained in DMEM with 10% FBS with or without IL-22 for 24 h.

### RNA interference

A small interfering RNA (siRNA) construct targeting the IL-17A was used with the transfection system to knockdown IL-17A expression. The set of siRNAs against IL-17A were obtained from GenePharma (Shanghai, China). The sequences of the siRNAs were available in Table 2. NIH/3T3 cells and MLFs were transfected with siRNAs and siRNA-Mate transfection reagent (GenePharma) according to the manufacturer’s recommendations.

**Table 2:** Sequences of siRNAs targeting IL-17A

SiRNA		Sequence (5′ to 3′)
IL-17A-1	Sense	CCAAGGACUUCCUCCAGAATT
	Antisense	UUCUGGAGGAAGUCCUUGGTT
IL-17A-2	Sense	GACCCUGAUAGAUAUCCCUTT
	Antisense	AGGGAUAUCUAUCAGGGUCTT
IL-17A-3	Sense	CUUUCAGGGUCGAGAAGAUTT
	Antisense	AUCUUCUCGACCCUGAAAGTT

### Statistics

Data were presented as mean ± standard error. Comparisons of means were performed with unpaired Student’s *t* test or one-way ANOVA test. *P*-values < 0.05 were considered statistically significant.

## Results

### IL-22 inhibits bleomycin-induced pulmonary fibrosis in mice

**Fig. 1:**
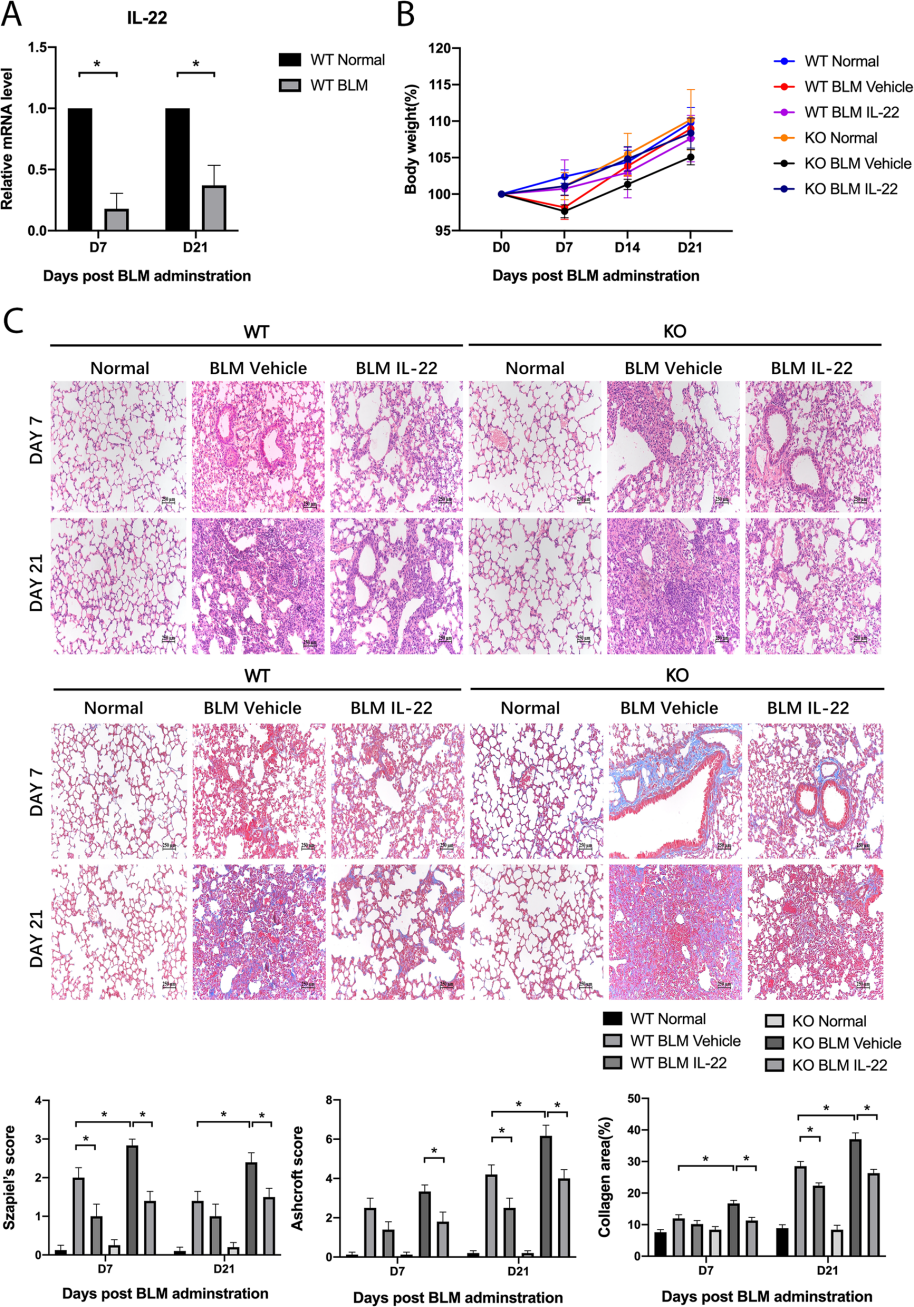
IL-22 inhibits bleomycin-induced pulmonary fibrosis. To induce pulmonary fibrosis, mice were intratracheally injected with bleomycin followed by treatments with vehicle or IL-22. Normal mice injected intraperitoneally with PBS were used as control. **A** Lung tissues were collected at day 7 and day 21 post bleomycin administration (*n* = 6). qPCR was used to detect mRNA level of IL-22 in lung tissues (*n* = 6). GAPDH was used as a normalization gene. Data represent fold changes relative to normal control. **B** Body weight was monitored continuously (*n* = 6). **C** H&E staining and Masson staining were performed on paraffin slides (*n* = 6). Szapiel’s score was used to evaluate alveolar inflammation and lung injury, Ashcroft score, and collagen deposition were analyzed. Scale bar: 250 μm. Data are mean ± SEM, *t*-test was used for comparison between two groups, and one-way ANOVA was used for comparison between multiple groups. *, *P* < 0.05

**Fig. 2:**
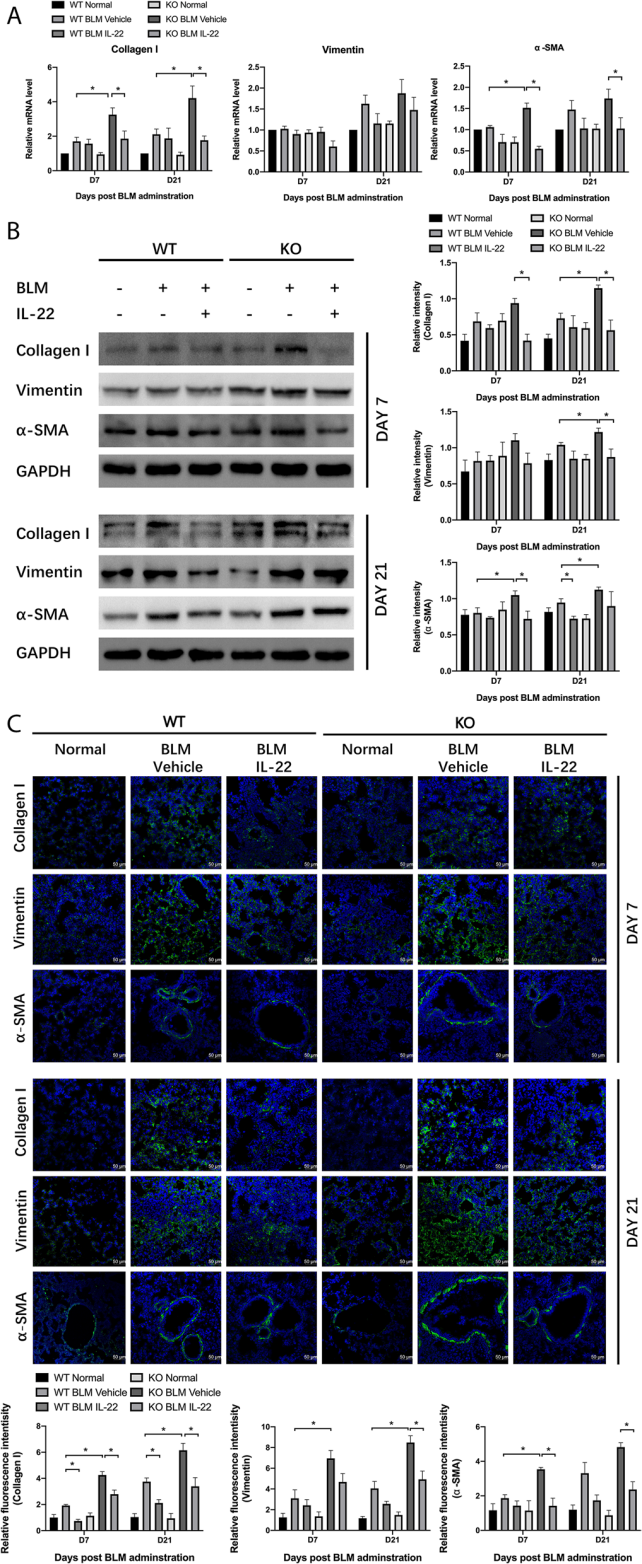
Impact of IL-22 on the expression of fibrosis-related genes. In the bleomycin-induced pulmonary fibrosis model, lung tissues were collected at day 7 and day 21. **A**, **B** qPCR and western blotting were used to detect mRNA (*n* = 6) and protein (*n* = 3) levels of Collagen I, vimentin, and α-SMA. GAPDH was used as a normalization gene. Relative intensity of each band was normalized to GAPDH protein. The relevant gels and blots were cropped. **C** Immunofluorescence was performed on cryosection of lung tissues with indicated antibodies (*n* = 3). Scale bar: 50 μm. Relative fluorescence intensity was analyzed. Data are mean ± SEM, compared using one-way ANOVA test. *, *P* < 0.05

To induce pulmonary fibrosis, WT C57BL/6 mice were intratracheally injected with bleomycin. According to our previous study[28], in the bleomycin-induced pulmonary fibrosis model, pulmonary inflammation was the main manifestation at day 7, and pulmonary fibrosis was obvious at day 21. In this study, we found that IL-22 mRNA levels decreased at day 7 and day 21 post bleomycin administration (Fig. 1A). Next, WT mice and IL-22KO mice were used in the pulmonary fibrosis model. IL-22KO mice showed aggravated pulmonary fibrosis compared with WT mice, and injection of recombinant IL-22 reversed the severe fibrotic manifestations in IL-22KO mice. Compared with WT mice, IL-22KO mice showed more weight lost (Fig. 1B) and increased pulmonary fibrosis as evidenced by increased Szapiel’s score and Ashcroft score (Fig. 1C). Next, we assessed the mRNA and protein levels of fibrosis-related genes including Collagen I, vimentin, and α-SMA. Bleomycin administration in IL-22KO mice significantly increased mRNA and protein levels of Collagen I, vimentin, and α-SMA in lung tissues, an effect reversed by recombinant IL-22 (Fig. 2A and B). Recombinant IL-22 also decreased histological changes as evidenced by Szapiel’s score and Ashcroft score (Fig. 1C). Immunofluorescence detection also showed knockout of IL-22 increased expressions of Collagen I, vimentin, and α-SMA in lung tissues and reversed by giving recombinant IL-22, which was consistent with the findings of mRNA and protein levels (Fig. 2C). Thus, IL-22 exerted an anti-fibrotic effect in the bleomycin model.

### IL-22 inhibits collagen production of A549 cells, NIH/3T3 cells, and MLFs *in vitro*

**Fig. 3:**
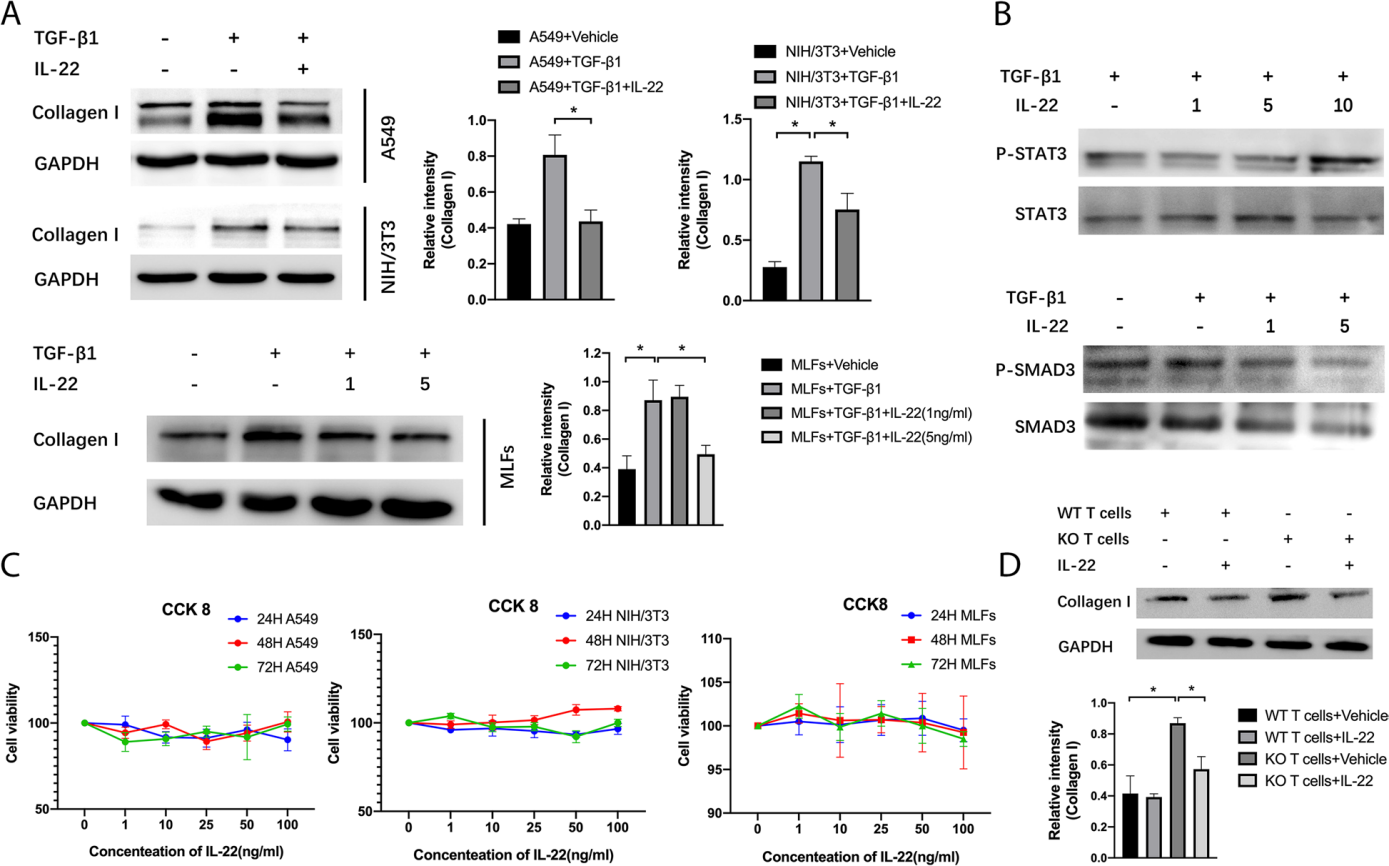
IL-22 inhibits collagen production of A549 cells, NIH/3T3 cells, and MLFs *in vitro*. **A** A549 cells and NIH/3T3 cells were cultured with 5 ng/ml TGF-β1 with or without IL-22 (1 ng/ml) for 48 h. MLFs were cultured with 5 ng/ml TGF-β1 with or without IL-22 (1 ng/ml, 5 ng/ml) for 48 h. Western blotting was used to detect proteins (*n* = 3). Relative intensity of each band was normalized to GAPDH protein. The relevant gels and blots were cropped. (B) MLFs were cultured with 5 ng/ml TGF-β1 with or without IL-22(1 ng/ml, 5 ng/ml, 10 ng/ml) for 48 h. Western blotting was used to detect proteins as indicated (*n* = 3). Relative intensity of each band was normalized to GAPDH protein. The relevant gels and blots were cropped. **C** A549 cells, NIH/3T3 cells, and MLFs were cultured with gradient doses of IL-22 for 24 h, 48 h, and 72 h in the presence of 5 ng/ml TGF-β1. Cell viability was measured using the CCK-8 assay (*n* = 6). Viability of cells without IL-22 treatment was set as 100%. **D** NIH/3T3 cells without TGF-β1 were co-cultured with T cells of WT or IL-22KO mice for 24 h with or without IL-22(10 ng/ml), and T cells were activated with CD3 and CD28. Relative intensity of each band was normalized to GAPDH protein. The relevant gels and blots were cropped. Data are mean ± SEM, compared using one-way ANOVA test. *, *P* < 0.05

Epithelial cells and fibroblasts are the main effector cells of pulmonary fibrosis. We analyzed the impact of IL-22 on Collagen I production of A549 cells, NIH/3T3 cells, and MLFs *in vitro*. TGF-β1 was used to induce Collagen I-expression in these cells[29–31], and IL-22 significantly decreased Collagen I protein levels in A549 cells, NIH/3T3 cells, and MLFs (Fig. 3A). IL-22 had no significant impact on the proliferation of TGF-β1-treated A549 cells, NIH/3T3 cells and MLFs at concentrations ranging from 0 to 100 ng/ml (Fig. 3C). These results indicated that IL-22 inhibited the production of Collagen I in A549 cells, NIH/3T3 cells and MLFs *in vitro*. Because IL-22 is mainly produced by T cells, we analyzed the effect of T cell-derived IL-22 on collagen production. NIH/3T3 cells, co-cultured with IL-22KO T cells, expressed a higher level of Collagen I protein compared with those co-cultured with WT T cells. Furthermore, adding IL-22 reduced the protein level of Collagen I in NIH/3T3 cells which were co-cultured with IL-22KO T cells (Fig. 3D), indicating that T cell-derived IL-22 inhibited collagen production in fibroblasts.

IL-22 induces STAT activation in several cell lines, such as mesangial cells, lung, and intestinal cells[32]. We found IL-22 significantly increased phosphorylation of STAT3 protein in MLFs (Fig. 3B). TGF-β1/SMAD signaling plays a significant role in promoting myofibroblast differentiation in mice with pulmonary fibrosis[33]. We found IL-22 decreased phosphorylation of SMAD3 protein *in vitro* (Fig. 3B).

### IL-22 mediated anti-fibrotic effect is associated with inhibition of IL-17A

**Fig. 4:**
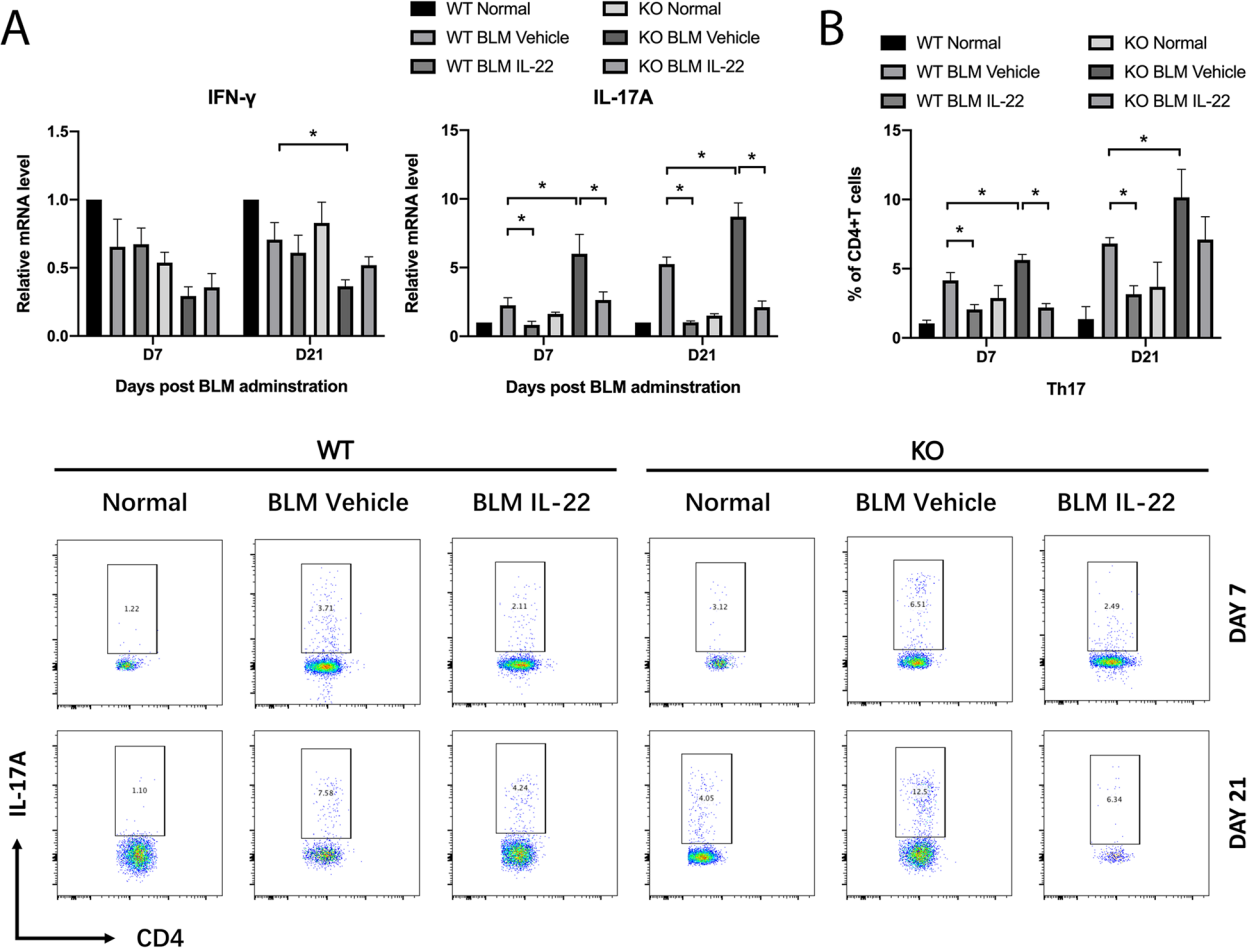
IL-22 decreases the production of IL-17A. **A** In the bleomycin-induced pulmonary fibrosis model, lung tissues were collected at day 7 and day 21. qPCR was used to detect mRNA (*n* = 6) levels of IL-17A and IFN-γ. **B** Flow cytometry was used to analyze the proportion of Th17 cells in lung tissues (*n* = 4) at day 7 and day 21 after bleomycin administration. Data are mean ± SEM, compared using one-way ANOVA test. *, *P* < 0.05

**Fig. 5:**
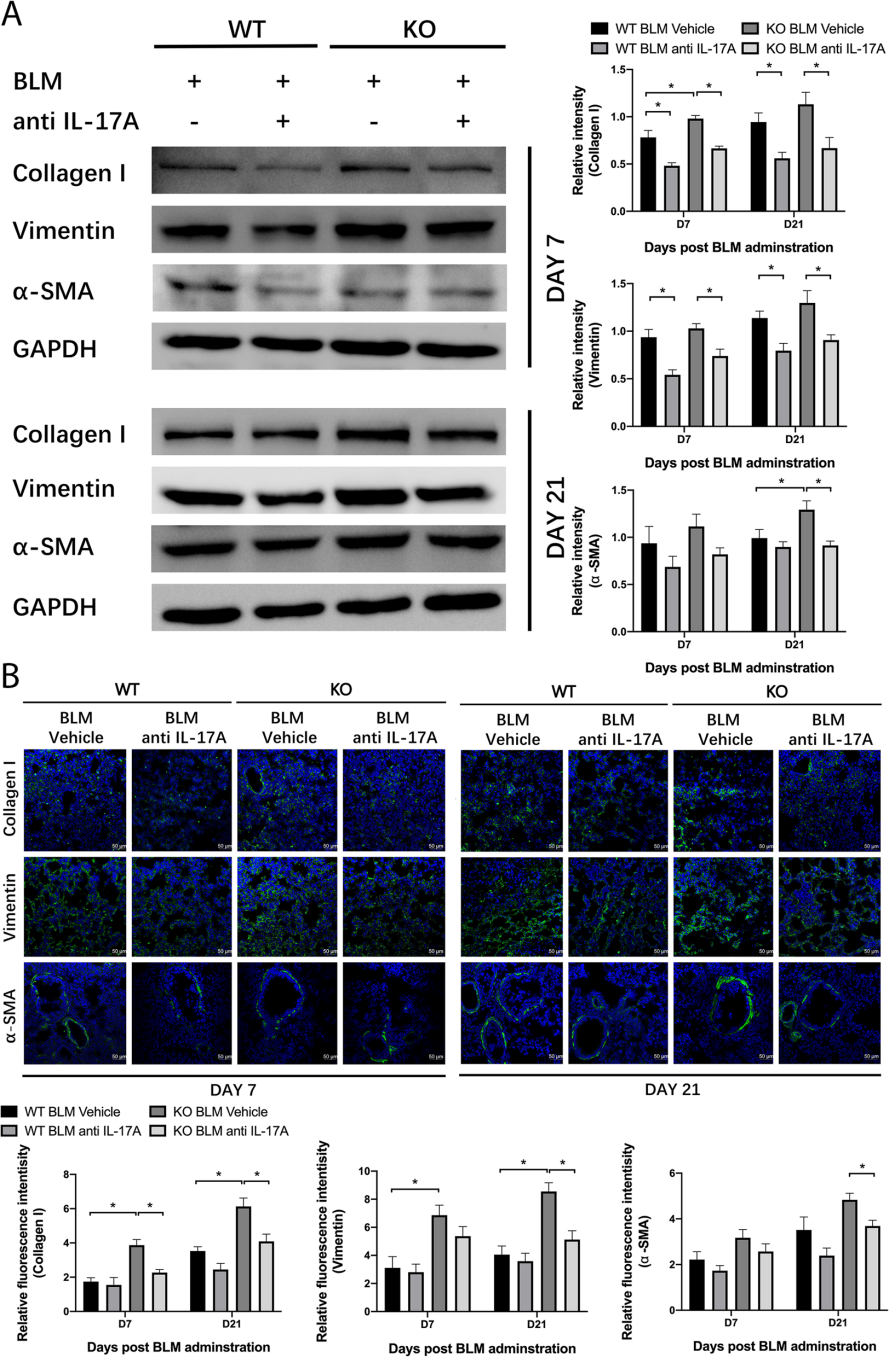
The IL-17A neutralizing antibody inhibits the expression levels of fibrosis-related genes. In the bleomycin-induced pulmonary fibrosis model, lung tissues were collected at day 7 and day 21. **A** Western blotting was used to detect protein (*n* = 3) levels of Collagen I, vimentin, and α-SMA. Relative intensity of each band was normalized to GAPDH protein. The relevant gels and blots were cropped. **B** Immunofluorescence was performed on cryosection of lung tissues with indicated antibodies (*n* = 3). Scale bar: 50 μm. Relative fluorescence intensity was analyzed. Data are mean ± SEM, compared using one-way ANOVA test. *, *P* < 0.05

**Fig. 6:**
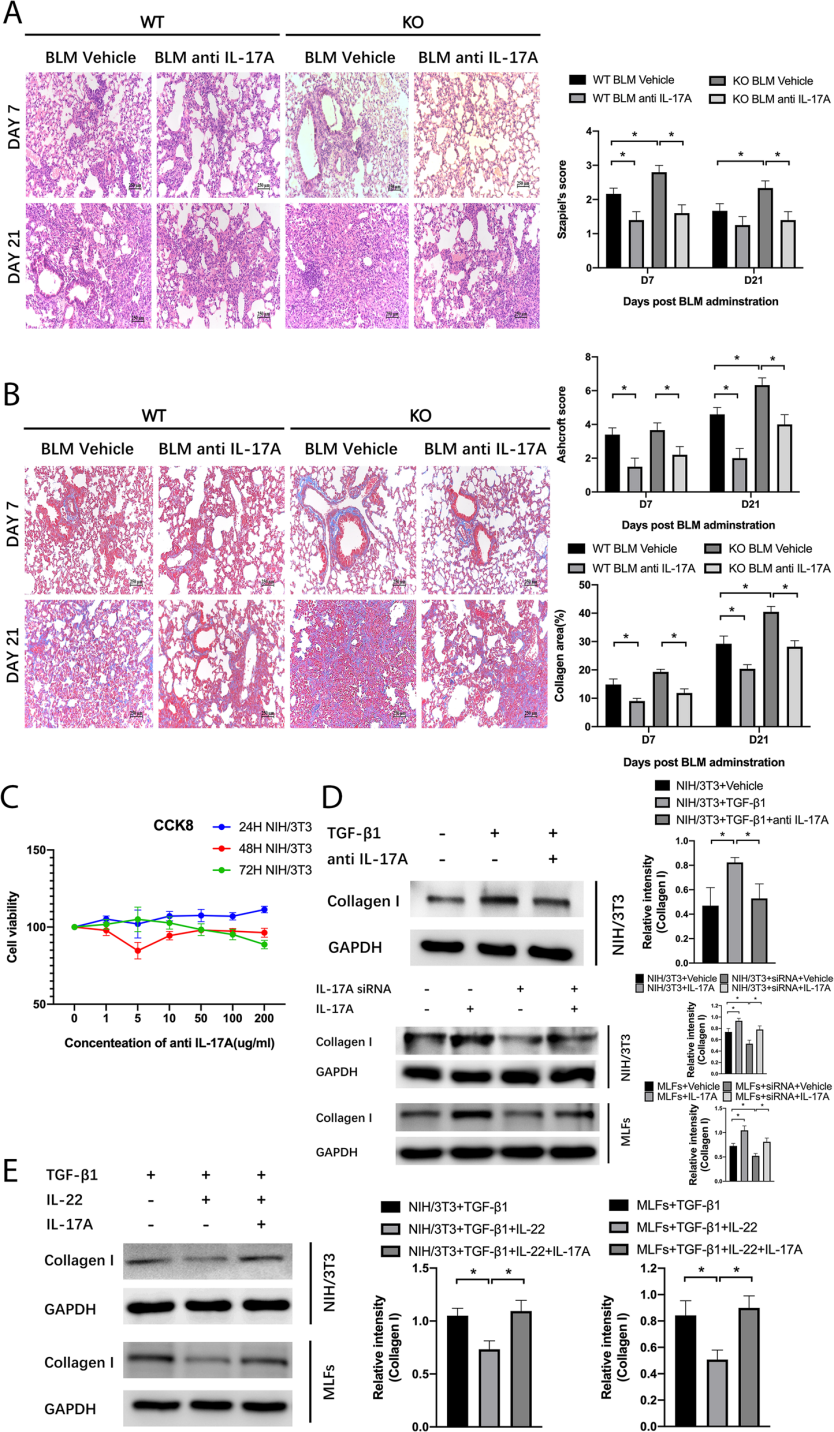
The IL-17A neutralizing antibody ameliorates severe pulmonary inflammation and fibrosis. **A** H&E staining was performed on paraffin slides (*n* = 6). Szapiel’s score was used to evaluate alveolar inflammation and lung injury. Scale bar: 250 μm. **B** Masson staining was performed on paraffin slides (*n* = 6). Ashcroft score and collagen deposition were analyzed. Scale bar: 250 μm. **C** NIH/3T3 cells were cultured with gradient doses of the IL-17A neutralizing antibody for 24 h, 48 h, and 72 h in the presence of 5 ng/ml TGF-β1. Cell viability was measured using the CCK-8 assay (*n* = 6). Viability of cells without the IL-17A neutralizing antibody treatment was set as 100%. (D) NIH/3T3 cells were cultured with 5 ng/ml TGF-β1 with or without the IL-17A neutralizing antibody (1 μg/ml) for 48 h. NIH/3T3 cells and MLFs without TGF-β1 were cultured in the presence of 150 pmol IL-17A siRNAs, 50 ng/ml IL-17A, or a combination of IL-17A siRNAs and IL-17A in six-well plates. Western blotting was used to detect proteins (*n* = 3). Relative intensity of each band was normalized to GAPDH protein. The relevant gels and blots were cropped. (E) NIH/3T3 cells were cultured with 5 ng/ml TGF-β1 with or without IL-22(1 ng/ml) and IL-17A (50 ng/ml) for 48 h. MLFs were cultured with 5 ng/ml TGF-β1 with or without IL-22(5 ng/ml) and IL-17A (50 ng/ml) for 48 h. Western blotting was used to detect proteins (*n* = 3). Relative intensity of each band was normalized to GAPDH protein. The relevant gels and blots were cropped. Data are mean ± SEM, compared using one-way ANOVA test. *, *P* < 0.05

To explore how T cells increase collagen production of NIH/3T3 cells, we assessed expressions of IL-17A and IFN-γ, two other mediators of pulmonary fibrosis. The mRNA level of IL-17A increased after bleomycin administration, especially in IL-22KO mice, and this effect was inhibited by concurrent use of IL-22, while the IFN-γ level was not significantly changed (Fig. 4A). We measured mRNA levels of other subtypes of IL-17 (Fig. S2), and found that the expression levels of these IL-17 subtypes were not significantly correlated with knockout of IL-22 or administration of IL-22. IL-22 also reduced the proportion of Th17 cells in lung tissues (Fig. 4B). Therefore, we hypothesized that IL-22 might function through inhibiting IL-17A. The IL-17A neutralizing antibody reduced protein levels of Collagen I, vimentin, and α-SMA at day 7 and day 21 post bleomycin administration in IL-22KO mice (Fig. 5A). Immunofluorescence detection also showed giving the IL-17A neutralizing antibody decreased expressions of Collagen I, vimentin and α-SMA in lung tissues of the bleomycin-treated mice (Fig. 5B). Consequently, the IL-17A antibody decreased bleomycin-induced pulmonary fibrosis as evidenced by decreased fibrosis-related Szapiel’s score, Ashcroft scores and reduced collagen deposition (Fig. 6A and B).

Next, we studied the effect of IL-17A *in vitro*. The IL-17A neutralizing antibody had no significant effect on the proliferation of TGF-β1-treated NIH/3T3 cells at concentrations ranging from 0 to 200 μg/ml (Fig. 6C). Protein level of Collagen I in NIH/3T3 cells decreased after intervention with the IL-17A neutralizing antibody *in vitro* (Fig. 6D). We also used IL-17A-targeted siRNAs to knock down expression of IL-17A in these fibroblasts (Fig. S3). IL-17A siRNAs decreased protein levels of Collagen I in NIH/3T3 cells and MLFs, an effect reversed by adding recombinant IL-17A (Fig. 6D). To test if IL-17A counteracted the effect of IL-22, we simultaneously used recombinant proteins IL-22 and IL-17A to treat NIH/3T3 cells and MLFs, and we found IL-17A increased protein levels of Collagen I which was decreased by IL-22 (Fig. 6E). These data showed IL-22-mediated anti-fibrogenesis might associate with the inhibition of IL-17A.

## Discussion

ILD is a common pulmonary complication of CTD patients including systemic sclerosis, Sjögren's syndrome, polymyositis/dermatomyositis, and rheumatoid arthritis, and is the main cause of death in these patients [34, 35]. Glucocorticoids and immunosuppressants are used to treat ILD in clinical practices, but with considerable side effects, and some patients have a poor therapeutic effect; thus, a new therapy is still needed [3, 36]. Pirfenidone and nintedanib have been approved for the treatment of idiopathic pulmonary fibrosis, but some patients are ineffective, and the effects of CTD-ILD treatment are inconsistently reported [37, 38]. The persistent chronic inflammation in the early stage of ILD causes the aggregation of inflammatory cells, including lymphocytes and macrophages, which release a large amount of inflammatory factors, stimulate proliferation and activation of fibroblasts, and secrete extracellular matrix, resulting in alveolar structure destruction and pulmonary fibrosis [39]. IL-22 is secreted by some immune cells and plays an important role in immune defense and immune homeostasis in the skin [40], digestive tract, and respiratory tract. IL-22 is a key cytokine in the regulation of inflammatory responses in the skin, mediating keratinocyte migration and differentiation and epithelial hyperplasia leading to epidermal remodeling [41]. Previous studies have shown a controversial role for IL-22 in fibrotic diseases, inducing pro-inflammatory responses as well as a tissue-protective effect. Our study showed that the mRNA level of IL-22 was decreased in bleomycin-induced pulmonary fibrosis mice, and pulmonary fibrosis was aggravated in IL-22KO mice, whereas the addition of exogenous recombinant IL-22 reduced pulmonary fibrosis-related genes, especially Collagen I, suggesting the potential protective effect of IL-22 in bleomycin-induced pulmonary fibrosis mice. We measured the IL-22RA1 mRNA level of lung tissues in normal mice and in bleomycin-induced pulmonary fibrosis mice. The IL-22RA1 mRNA level increased after bleomycin treatment. Therefore, giving IL-22 after bleomycin administration is a reasonable time point. IL-22 also reduced the protein level of Collagen I in A549 cells, NIH/3T3 cells, and MLFs, further confirming the results *in vivo*. Furthermore, to explore the mechanism of the IL-22-mediated anti-fibrogenesis effect, we detected the proportion of Th17 cells in lung tissue by flow cytometry. We found IL-22 reduced the proportion of Th17 cells and the IL-17A mRNA level, suggesting that IL-22 might function through inhibiting IL-17A.

IL-10 is a well-known anti-inflammatory cytokine, mainly produced by macrophages and lymphocytes [42, 43]. IL-10 has a protective effect in acute inflammatory diseases such as asthma and acute respiratory distress syndrome, but the role of IL-10 in pulmonary fibrosis is unclear [44]. Studies have shown that IL-10 has a therapeutic effect in pulmonary fibrosis [45]. IL-22 is a member of the IL-10 cytokine family and has 23% homology with IL-10 in structure [9]. IL-22 plays an important role in both innate and adaptive immunity and participates in the pathophysiological process of various lung tissues. In mice infected with Klebsiella pneumoniae, IL-22 can promote epithelial repair, inhibit epithelial damage, and thus inhibit the inflammatory response; in mice infected with chlamydia pneumoniae, IL-22 can participate in host defense against chlamydia infection by regulating Th1/Th17 [46]; the expression of IL-22 was decreased in bleomycin-induced pulmonary fibrosis in mice [47], indicating that IL-22 may have a protective effect, which is consistent with our conclusion that IL-22 can inhibit the development of pulmonary fibrosis.

IL-22 was originally found to be produced by T cells and showed a relation with Th17 cells [20, 21]. IL-17A, mainly produced by Th17 cells, induces the expression of pro-inflammatory cytokines such as TNF-α and IL-1β and promotes neutrophil migration [48]. IL-17A is involved in respiratory diseases such as bronchial asthma and can promote collagen formation and pulmonary fibrosis. However, the numbers of lymphocytes, monocytes, and neutrophils in the bronchoalveolar lavage fluid of mice decreased after injection of IL-17 neutralizing antibody, indicating that IL-17 neutralizing antibody can reduce the inflammatory response [49]. Our study also confirmed that giving the IL-17A neutralizing antibody decreased pulmonary fibrosis and collagen deposition in both WT mice and IL-22KO mice. The IL-17A mRNA level increased but the IL-22 mRNA level decreased in bleomycin-treated mice. Using exogenous recombinant IL-22 reduced IL-17A mRNA levels in lung tissues. These results indicated balance between IL-22 and IL-17A might be important for controlling pulmonary fibrosis in the bleomycin model.

It has been reported that IL-17 can activate the STAT3 signaling pathway to produce collagen [50], and we found that the IL-17A neutralizing antibody can reduce collagen expression in NIH/3T3 cells. CD4^+^T cells not only induce apoptosis of host cells, but also secrete pro-fibrotic cytokines, especially TGF-β and IL-1β, which activate fibroblasts and myofibroblasts, leading to collagen deposition and tissue remodeling [51]. Inflammatory factors secreted by Th17 cells can directly stimulate the proliferation of fibroblasts, secrete more pro-fibrotic factors, and further stimulate fibroblast proliferation and collagen deposition [52, 53]. We found that IL-22 reduced the protein level of Collagen I in NIH/3T3 cells which were co-cultured with T cells, indicating that IL-22 could inhibit the profibrotic factor secreted by T cells. Other actions might also contribute to IL-22-mediated anti-fibrogenesis. It has also been studied that the anti-fibrotic effect of IL-22 may directly inhibit the TGF-β/SMAD pathway[54]. We also found IL-22-mediated anti-fibrogenesis might associate with activating JAK/STAT3 pathway and inhibiting TGF-β/SMAD3 pathway.

Our study has limitations. Using primary lung fibroblasts from diseased humans would better interpret the function of IL-22 and related inflammatory cytokines, and bleomycin-induced fibrosis may not fully reflect the complicated background of diseased humans.

## Conclusions

In conclusion, we found IL-22 mediated an anti-fibrogenesis effect in the bleomycin-induced pulmonary fibrosis model and the effect associated with inhibition of IL-17A. IL-22 may serve as a potential target for intervening pulmonary fibrosis.

## Supplementary Information

**Additional file 1: Table S1.** Sequence of primers used in the study. **Figure S1.** Lung tissues were collected at day 7 post bleomycin administration. qPCR was used to detect mRNA level of IL-22RA1 in lung tissues (*n* = 6). GAPDH was used as a normalization gene. Data represent fold changes relative to normal control. **Figure S2.** In the bleomycin-induced pulmonary fibrosis model, lung tissues were collected at day 7 and day 21. qPCR was used to detect mRNA (*n* = 6) levels of IL-17 subtypes. Data are mean ± SEM, compared using one-way ANOVA test. *, *P* < 0.05. **Figure S3.** NIH/3T3 cells and MLFs were cultured with or without the IL-17A siRNAs. Western blotting was used to detect protein level of IL-17A (*n* = 3). Relative intensity of each band was normalized to GAPDH protein. The relevant gels and blots were cropped. Data are mean ± SEM, compared using t-test. *, *P* < 0.05.

## Data Availability

The data that support the findings of this study are available from the corresponding author on reasonable request.

## Abbreviations

IL-22: Interleukin-22
CTD: Connective tissue disease
ILD: Interstitial lung disease
MMP: Matrix metalloproteinase
TIMP: Tissue inhibitor of metalloproteinase
WT: Wild-type
KO: Knockout
FBS: Fetal bovine serum
α-SMA: Alpha‐smooth muscle actin
siRNA: Small interfering RNA
MLFs: Mouse lung fibroblasts
