# Supplementary material for: IL-22 inhibits bleomycin-induced pulmonary fibrosis in association with inhibition of IL-17A in mice
Source: Arthritis Res Ther. 2022 Dec 24;24:280. doi: 10.1186/s13075-022-02977-6 (PMC9789559; doi:10.1186/s13075-022-02977-6)
Supplement: Supplementary file 1 — Additional file 1: Table S1. Sequence of primers used in the study. Figure S1. Lung tissues were collected at day 7 post bleomycin administration. qPCR was used to detect mRNA level of IL-22RA1 in lung tissues (n = 6). GAPDH was used as a normalization gene. Data represent fold changes relative to normal control. Figure S2. In the bleomycin-induced pulmonary fibrosis model, lung tissues were collected at day 7 and day 21. qPCR was used to detect mRNA (n = 6) levels of IL-17 subtypes. Data are mean ± SEM, compared using one-way ANOVA test. *, P < 0.05. Figure S3. NIH/3T3 cells and MLFs were cultured with or without the IL-17A siRNAs. Western blotting was used to detect protein level of IL-17A (n = 3). Relative intensity of each band was normalized to GAPDH protein. The relevant gels and blots were cropped. Data are mean ± SEM, compared using t-test. *, P < 0.05. [file 13075_2022_2977_MOESM1_ESM.docx]

**supplemental information**

**Table S1** Sequence of primers used in the study

| Gene | Forward (5’-3’) | Reverse (5’-3’) |
| --- | --- | --- |
| IL-22RA1 | CAGCGGATCACCCAGAAGTT | GCGGTTTGATGGTAGTGTGC |
| IL-17B | GAACCTTGGGGAGATGGTGG | GGGTCGTGGTTGATGCTGTA |
| IL-17C | ACCATGGAGATATCGCATCGAC | GTCGCCGTAGTACCAGCAG |
| IL-17D | CTTCTGTAGGGGCGACACC | CGCGAGCATCCAGACCAGT |
| IL-17E | TCCTTGGAGCTATGAGTTGGAC | TGTGGGAGCCTGTCTGTAGG |
| IL-17F | GAAGGCTGGGAACTGTCCTC | GGGGTCTCGAGTGATGTTGT |


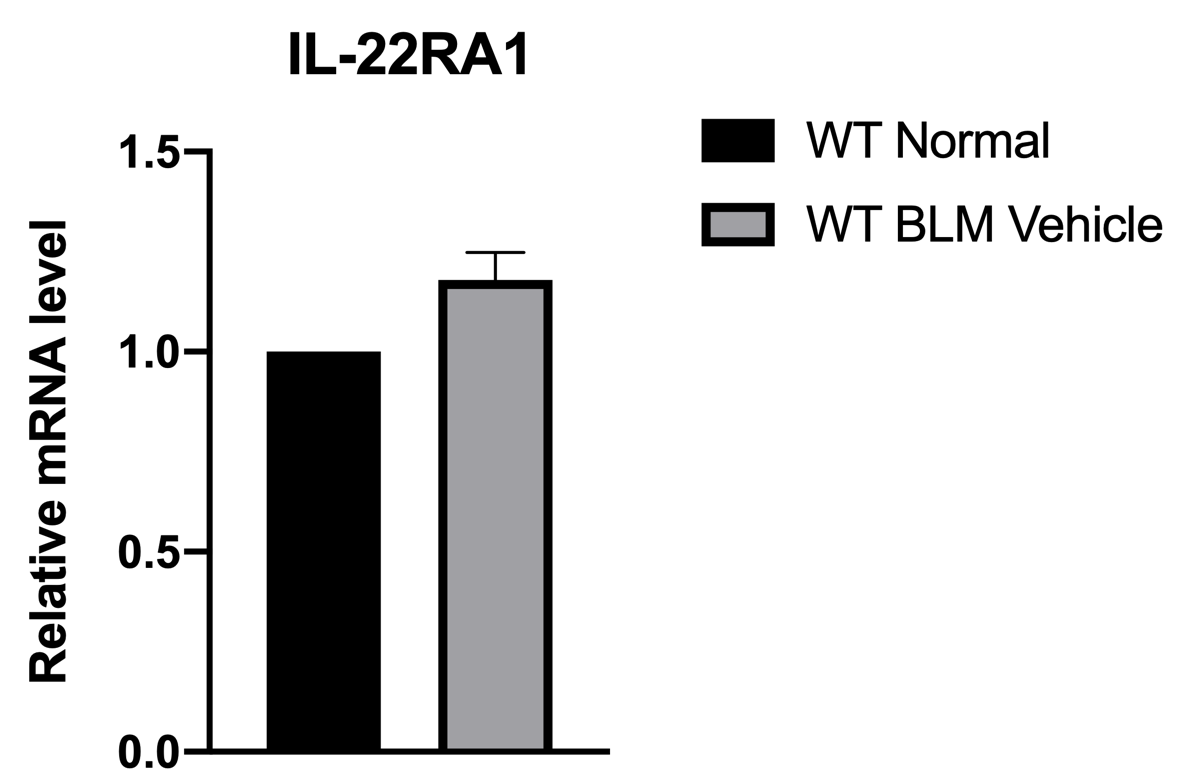


**Figure S1.** Lung tissues were collected at day 7 post bleomycin administration. qPCR was used to detect mRNA level of IL-22RA1 in lung tissues (n = 6). GAPDH was used as a normalization gene. Data represent fold changes relative to normal control.


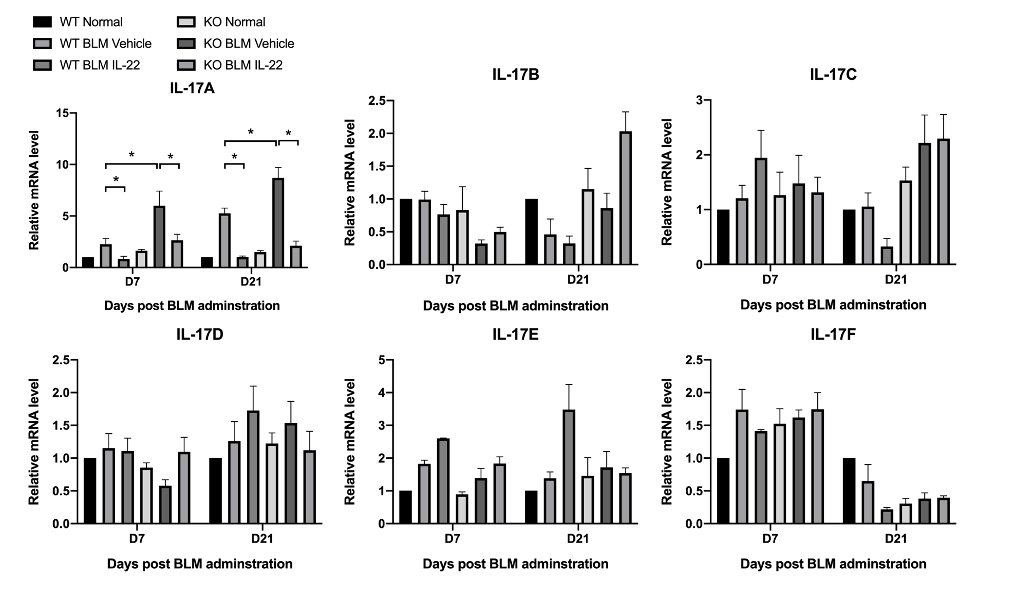


**Figure S2.** In the bleomycin-induced pulmonary fibrosis model, lung tissues were collected at day 7 and day 21. qPCR was used to detect mRNA (n = 6) levels of IL-17 subtypes. Data are mean ± SEM, compared using one-way ANOVA test. *, P < 0.05.


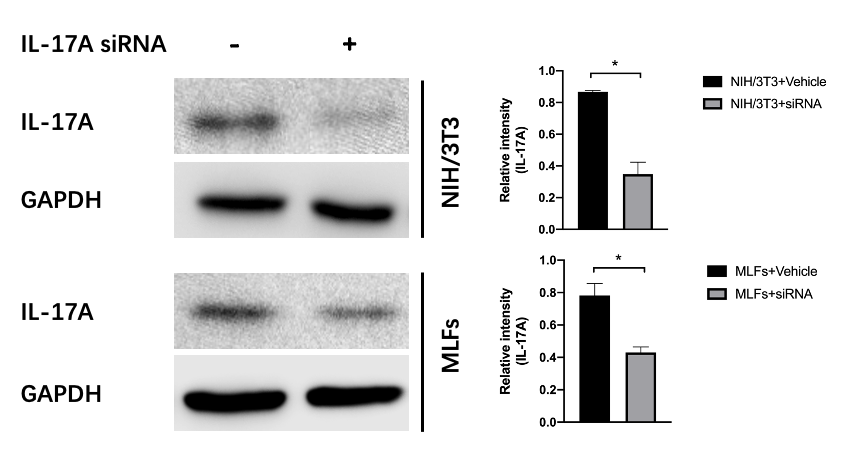


**Figure S3.** NIH/3T3 cells and MLFs were cultured with or without the IL-17A siRNAs. Western blotting was used to detect protein level of IL-17A (n = 3). Relative intensity of each band was normalized to GAPDH protein. The relevant gels and blots were cropped. Data are mean ± SEM, compared using t-test. *, P < 0.05.
